# Metastability of resting-state bold fMRI as a reliable biomarker of individual brain dynamics: An interrogation of within-subject variability as a function of total acquisition time

**DOI:** 10.1162/NETN.a.537

**Published:** 2026-04-22

**Authors:** Hiba Sheheitli, Robert Hermosillo, Gracie Grimsrud, Thomas Madison, Oscar Miranda Dominguez, Steven Nelson, Damien Fair, Ziad Nahas

**Affiliations:** Department of Psychiatry and Behavioral Sciences, University of Minnesota, Minneapolis, USA; Department of Neurology, University of Minnesota, Minneapolis, USA; Department of Pediatrics, University of Minnesota, Minneapolis, USA; Masonic Institute for the Developing Brain, University of Minnesota, Minneapolis, USA; Developmental Cognition and Neuroimaging Lab, University of Minnesota, Minneapolis, USA; Minnesota Supercomputing Institute, University of Minnesota, Minneapolis, USA; Institute of Child Development, University of Minnesota, Minneapolis, USA

**Keywords:** Metastability, Phase synchrony, Brain dynamics biomarker, Dynamic functional connectivity, BOLD fMRI resting-state networks, Precision functional mapping

## Abstract

Metastability of BOLD fMRI signals is a commonly used proxy of brain dynamics in behavioral and clinical studies. To date, little has been done to assess the confidence with which we can use estimates of metastability as reliable biomarkers of individual brain state. We analyze whole-brain and network-specific metastability for a highly sampled individual brain (84 sessions taken over 18 months) and quantify the within-subject reliability for the metrics as a function of the amount of data used, which we find to be comparable to that seen for static functional connectivity. As considerable variability is observed across networks in the required amount of data, we combine the networks’ metrics in one novel feature vector that exhibits an order of magnitude improvement in reliability. We then test reproducibility by analyzing the Midnight Scan Club dataset (10 subjects imaged over 10 consecutive days). Finally, we examine the susceptibility to change of the proposed metastability measure in another dataset examining brain dynamics under the effect of psilocybin. We conclude that the networks’ metastability feature vector exhibits strong within-subject reliability that renders it a promising candidate for the study of individual-specific biomarkers of brain dynamics and potential targets for precision neuromodulation.

## Author Summary

Metastability reflects to a system’s tendency to spontaneously and intermittently switch between patterns of activity. While brain metastability measures have been associated with behavioral and clinical outcomes, their within-subject variability, particularly as a function of acquisition time, remains poorly characterized. We leveraged two unique datasets, a highly sampled individual repeatedly scanned over 18 months and nine individuals scanned over 10 days, to quantify the reliability of a standard proxy of metastability. Our novel feature vector, combining network-specific synchrony and metastability, exhibits an order-of-magnitude improvement in within-subject reliability, as compared with scalar measures and static functional connectivity. The metric showed sensitivity to psilocybin-induced changes while maintaining stability across sessions. Our findings suggest metastability could become a valuable biomarker for precision neuromodulation medicine in neurology and psychiatry.

## INTRODUCTION

Numerous psychiatric and neurological diseases are known to be associated with disturbances in neural dynamics, reflected in alterations in the spatiotemporal organization of brain oscillatory signals observed using different recording modalities (Günther & Hanganu-Opatz, 2022; Mathalon & Sohal, 2015; Uhlhaas & Singer, 2012). The technological advancement of noninvasive BOLD fMRI, with its high spatial resolution, has thus fueled the quest for “biomarkers” that reflect such dynamics and can serve as informative quantitative measures of brain state (Damoiseaux, 2012; Parkes et al., 2020; Woodward & Cascio, 2015; Yamada et al., 2017).

One measure of brain dynamics that is well positioned to serve this role is “metastability,” which has been invoked in numerous works to capture the nature of fluctuations of brain functional connectivity (FC), its alteration in disease, as well as its correlation to behavioral and clinical outcomes (Kelso, 1999; Tognoli & Kelso, 2014; see Hancock et al., 2025, for a recent thorough review).

**Figure 1.**
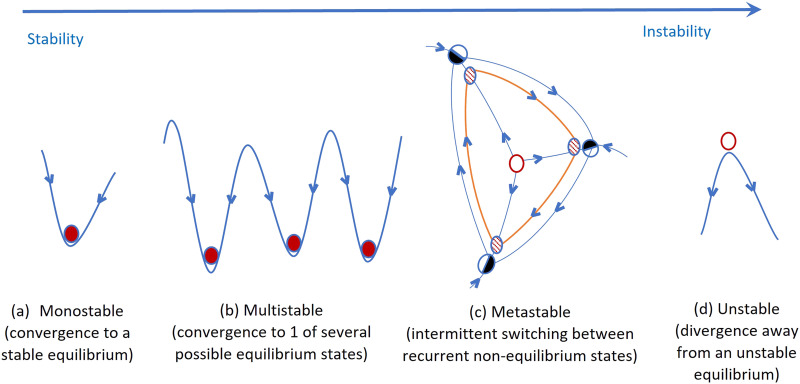
(Adapted from Hancock et al., 2025) Schematic illustration of different types of stability. (A) Monostable: The system approaches a single equilibrium point (red circle) for all initial conditions. (B) Multistable: The system can approach a finite number of equilibrium points for different initial conditions. (C) Metastable: The system approaches a finite number of “saddle” equilibrium points (half-filled black circles), along certain directions in state space, and is repelled along different directions, leading to trajectories (in orange) that intermittently revisit a finite number of states (dashed filled circles). (D) Unstable: The system diverges away from an unstable equilibrium point (unfilled red circle). Note that A, B, and D depict a side view perspective of an energy landscape of typical 2D systems, while C depicts a top view perspective of trajectories in state space of a high-dimensional system.

Metastability refers to a complex system's tendency to spontaneously and intermittently switch between a finite number of recurring states (Figure 1 illustrates metastability in contrast to other types of dynamics). Here, in the context of brain dynamics, “state” refers to the instantaneous level of phase synchrony among brain parcels; metastability is then quantified as the variability of that state of synchrony across time. This operationalization builds on the mathematical *Theory of Coordination Dynamics* (TCD; Tognoli & Kelso, 2009, 2014), which proposes that complex spatiotemporal brain dynamics can be understood as the manifestation of the coordinative “acting in concert” behavior of neuronal ensembles; brain metastability is thus postulated to reflect a balancing tug-of-war between the dual poles of *integration* (tendencies for neural ensembles to synchronize and work together) and *segregation* (tendencies for neural ensembles to desynchronize and function independently).

TCD builds on studies of the collective behavior of electrophysiological signals (EEG and EcoG) recorded across different spatial locations in the brain (Freeman & Holmes, 2005; Kozma & Freeman, 2008). When such signals are band-pass filtered into oscillatory components within specific frequency bands, it is observed that the trajectories of the phases of these signals tend to exhibit transiently formed grouping of neuronal ensembles that synchronize, and desynchronize, intermittently; this self-organization, which is a product of the brain's nonlinearity, high dimensionality, and multiscale nature, is thought to underlie the concurrent complexity and flexibility required for its magnificent functioning (Kelso, 1999). These earlier works on metastability in electrophysiological signals paved the way for the extension of these ideas into the analysis of BOLD fMRI signals, particularly with the discovery of the resting-state brain functional networks and the development of the computational whole-brain network modeling (Deco & Kringelbach, 2016; Deco et al., 2017; Ponce-Alvarez et al., 2015); the latter has played a pivotal role in providing theoretical reasoning to support the rationale that coordination dynamics underlying microscopic neuronal oscillatory interactions are indeed reflected at the macroscopic whole-brain level of slow oscillations observed in fMRI signals and, that consequently, metastability measures can be applicable to the analysis of BOLD fMRI.

A careful examination of the literature on metastability in BOLD fMRI brings to light a great variability across studies at the level of the technical details involved in the analysis, such as the choice of spatial resolution (e.g., brain parcellation for voxel grouping), motion artifact attenuation, the functional networks assignment method, the length of data acquisition, and the choice of frequency band for signal phase extraction, to name a few. Our work here is motivated by the need for a systematic approach to characterize parameters required for stable and repeatable measurements of metastability that can serve as reliable metrics for understanding brain diseases and responses to interventions.

More specifically, we aim to quantify the within-subject variability of a standard proxy measure of metastability as a function of the total signal acquisition duration. We believe this to be a critical evaluation to quantify the confidence with which we can use standard proxies of metastability as reliable indicators of an individual's brain state, a prerequisite for the designation of any informative or predictive biomarker in diagnostic and therapeutic intervention contexts.

This investigation can be seen as a natural extension of the seminal work in Laumann et al. (2015), which warned that the measure of *static* FC for a given subject can exhibit significant variability when the duration of recording session is not long enough and, consequently, has recommended that studies ought to use at least 100 min of data for reliable results. We here build on that approach to address the equivalent question for *dynamic* FC, more specifically, as captured by a standard measure of metastability.

The approach we follow for characterizing network dynamics, through instantaneous phase synchrony and metastability, differs from other commonly used dynamic FC methods in several key ways. Sliding window approaches estimate FC within successive temporal windows (typically 30–60 s), capturing slow fluctuations in correlation structure but with inherent trade-offs between temporal resolution and statistical reliability of correlation estimates (Allen et al., 2014; Hutchison et al., 2013). Change point detection methods identify discrete transitions between quasistable connectivity states, assuming a piecewise stationary model of brain dynamics (Cribben et al., 2012; Xu & Lindquist, 2015). In contrast, our instantaneous phase-based approach captures moment-to-moment fluctuations in network coordination without imposing arbitrary window lengths or assuming discrete states. By deriving narrowband signals through empirical mode decomposition (EMD) and computing instantaneous phase via the Hilbert transform, we obtain continuous time series of synchronization that preserve the natural temporal structure of neural dynamics. The metastability metric then quantifies the temporal variability of this synchronization, reflecting the flexibility with which networks transition between coordinated and desynchronized states. This framework aligns with theoretical perspectives emphasizing the importance of both integration (synchrony) and temporal flexibility (metastability) for adaptive brain function, while avoiding some of the methodological challenges associated with windowed correlation approaches, such as sensitivity to window length selection and the requirement for stationarity within windows.

We analyze the highly sampled individual (HSI) brain dataset (Laumann et al., 2015; Poldrack et al., 2015), in which one individual was studied over more than a year, accumulating 14 hr of resting-state fMRI data; we use the Gordon parcellation (Gordon et al., 2016; Gordon, Laumann, Adeyemo, & Petersen, 2017) to generate 333 parcel time series from which we compute the mean phase synchrony (sync) and corresponding metastability measure for the individual resting-state functional networks. We study the within-subject reliability, as a function of acquisition time, for the sync and metastability values of the functional networks taken individually; observing marked differences in the performance across the different networks, we propose a novel feature vector that combines values of the sync and metastability of all the individual functional networks and exhibits a significantly more robust within-subject reliability profile.

For further validation, we repeat the analysis using another unique dataset, known as the Midnight Scan Club (MSC) dataset (Gordon, Laumann, Gilmore, et al., 2017). With 30 min recording sessions repeated over 10 consecutive days per subject, the MSC dataset allows us to look at across-subject variability of the within-subject reliability results found using the HSI dataset. Finally, we examine the sensitivity of the metastability measure to strong changes in brain state by analyzing a subset of the data presented in Siegel et al. (2024) in which brain FC was probed under the effect of psilocybin and methylphenidate.

In what follows, we present the detailed methods and results and discuss the implications and relevance of the proposed framework. Before presenting our findings, we briefly clarify our key metrics. *Synchrony (Sync)* quantifies the average degree of phase alignment between brain regions within a network at each moment in time, reflecting the instantaneous level of coordination. *Metastability* measures the temporal variability of this coordination, quantified as the standard deviation of the instantaneous synchrony time series, capturing how flexibly networks transition between synchronized and desynchronized states. Networks with high metastability exhibit large fluctuations in coordination strength over time, while low metastability indicates more stable coordination patterns. *Sync-metastability* refers to a combined feature vector that concatenates the synchrony and metastability values across all functional networks (e.g., for 14 networks, this creates a 28-dimensional vector with 14 synchrony values followed by 14 metastability values). This joint representation captures both the average coordination strength and temporal flexibility profile across the brain's functional architecture. As we will demonstrate, this combined metric exhibits superior reliability compared with either measure alone or to traditional static FC. Ultimately, it can be seen that the combined sync-metastability feature vector exhibits significant within-subject reliability that renders the metric a promising candidate to serve as an informative biomarker of brain state for use in future mathematical modeling and clinical applications.

## RESULTS

### The Highly Sampled Brain

Work by Laumann and colleagues (2015) set out to quantify the amount of data necessary to reliably compute individual static FC; the approach consists of splitting the data in half, using 420 min to compute what can be considered as a “ground truth” or “gold standard” baseline FC and then computing its correlation to that obtained with only a subset of the remaining 420 min of data. One thousand iterations are done in which each iteration consists of a different random split of the data, such that for each amount of data used for the subset sample, we can compute the average and standard deviation of the obtained correlation values across the 1,000 iterations. The result is a convergence profile that quantifies the variability relative to the baseline given a certain amount of data used (in minutes) to compute the FC. The analysis ultimately demonstrated that at least 30 min of low motion data is required to achieve a 0.90 correlation to the “ground truth” baseline, as presented in Figure 2B in Laumann et al. (2015).

**Figure 2.**
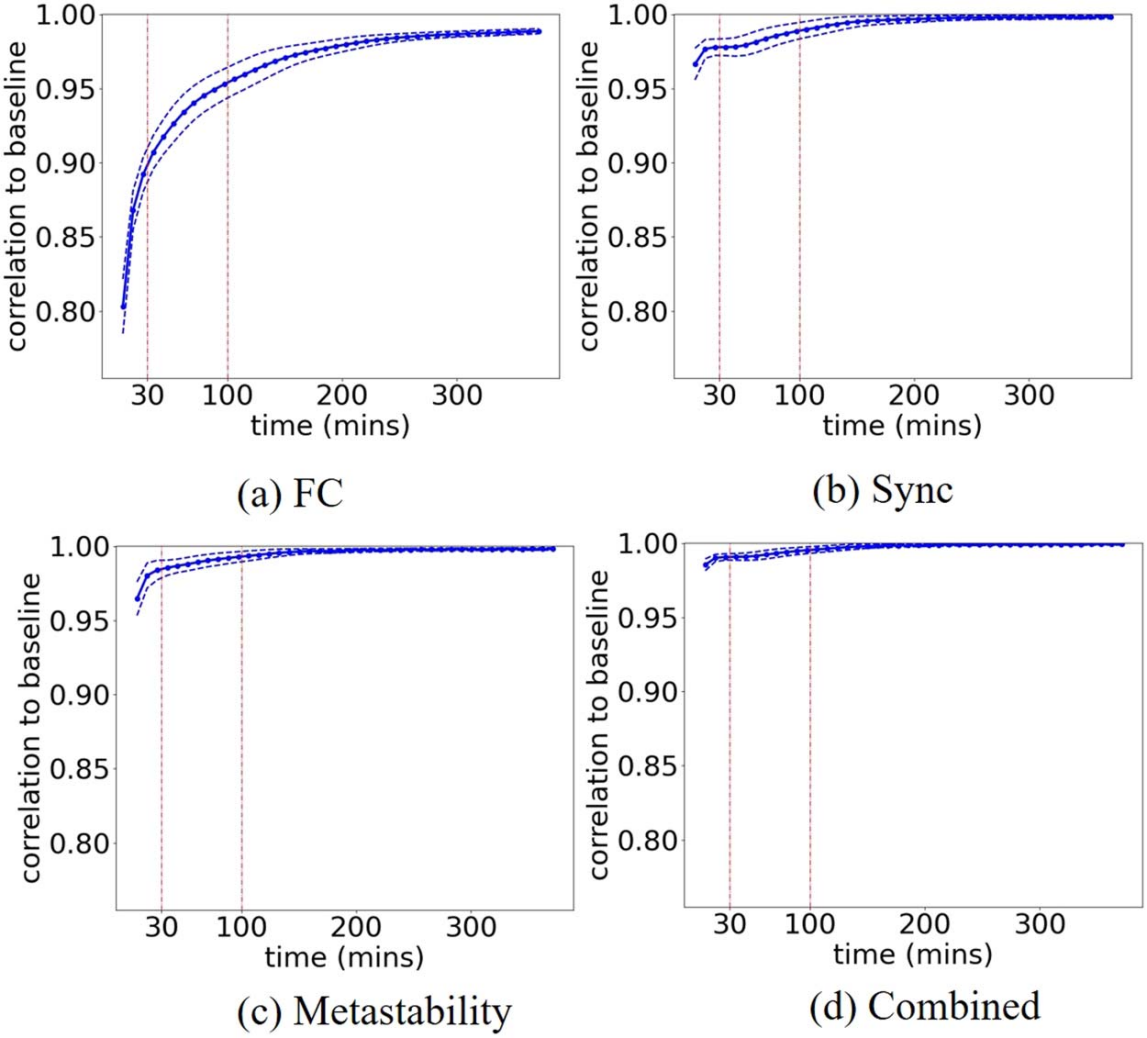
The average (solid line) correlation to baseline versus the total acquisition time for the static FC (A), sync feature vector (B), metastability feature vector (C), sync and metastability combined in one feature vector; (D) dashed lines show ± standard deviation.

We implement the same said approach and start by confirming these findings using our analytical pipeline applied to the HSI dataset, before extending the same approach to examine the reliability of sync and metastability metrics. Figure 2A shows the consistent result we obtained, confirming an average correlation close to 0.892 ± 0.012 (95% confidence interval (CI) [0.8914, 0.8929]) for 30 min of sample data that increases to 0.953 ± 0.010 (95% CI [0.9522, 0.9535]) for 100 min. Figure 2B shows the results of applying that same analysis to the sync feature vector containing the values of mean phase synchrony within each of the 14 individual networks, computed using the leading oscillatory components of the parcel activity time series. We performed the analysis for the two leading oscillatory components extracted via EMD, which we refer to as “mode 0” and “mode 1.” Mode 0 represents the fastest intrinsic oscillation (median frequency = 0.04–0.07 Hz), corresponding closely to the canonical low-frequency BOLD fluctuations commonly studied in resting-state fMRI (Glerean et al., 2012). Mode 1 represents slower oscillations (median frequency = 0.01–0.04 Hz), capturing dynamics at an extended temporal scale. By examining both modes, we assess whether metric reliability properties differ across these functionally distinct timescales of brain activity. We present results for mode 0 in the main text and mode 1 results in supplementary materials (see the Methods section for full details).

The resulting correlation to baseline converges faster than that for FC, reaching more than 0.978 ± 0.006 (95% CI [0.9776, 0.9783]) for 30 min of data; this is true for sync computed on both timescales, that for mode 0 in Figure 2B and mode 1 in Supporting Information Figure S1a.

The correlation to baseline shows a comparable profile for the metastability feature vector consisting of the metastability values of the individual networks, also on both timescales, mode 0 in Figure 2C and mode 1 in Supporting Information Figure S1b. The correlation to the baseline is significantly enhanced when the sync and metastability values are combined into one feature vector, as seen in Figure 2D and Supporting Information Figure S1c, reaching close to 0.991 ± 0.002 (95% CI [0.9906, 0.9909]) for 30 min of data; Supporting Information Figure S4 shows a zoomed-in view on Figures 2C–D.

We quantify the practical significance of reliability improvements by computing effect sizes comparing synchrony, metastability, and combined sync-metastability metrics to FC across data durations. At 8.8 min of data acquisition, sync-metastability reliability (*r* = 0.985 ± 0.004) significantly exceeded FC reliability (*r* = 0.803 ± 0.018) by 0.182 (paired *t*(999) = 306.64, *p* < 0.001, Cohen's *d* = 9.70), representing a 22.7% improvement and 92% reduction in unexplained variance. Synchrony (*r* = 0.966, Cohen's *d* = 7.66 vs. FC) and metastability (*r* = 0.964, Cohen's *d* = 7.48 vs. FC), individually, also showed substantial improvements over FC. Effect sizes remained very large (across all data durations tested [Cohen's d ranging from 9.70 at 8.8 min to 6.16 at 371 min for sync-metastability vs. FC]), demonstrating consistent practical advantages of the combined metric for characterizing individual brain dynamics, with particularly pronounced benefits at shorter scan durations commonly used in clinical and research settings.

To address potential concerns about redundancy between network-specific and global metrics, we examined the cross-correlations between all synchrony and metastability values (Supporting Information Figures S7 and S8). The mean absolute pairwise correlation among network-specific synchrony values converged to approximately 0.2 at baseline (370 min), with similar values observed for metastability metrics. We also find an average absolute correlation of ~0.22 and ~0.24 when looking specifically at the pairs that include global sync and global metastability, respectively. These modest correlations indicate that individual networks exhibit largely independent dynamics, supporting the inclusion of multiple networks in the feature vector without substantial risk of overfitting. The relatively weak correlations also suggest that each network contributes complementary information about brain state rather than redundant measurements of a common signal, which is consistent with the fact that combining metrics across networks substantially improves reliability.

Moreover, in order to confirm that the computed synchrony and metastability metrics capture genuine neural signal rather than statistical artifacts, we compared empirical metric values against phase-randomized surrogate data that preserve spectral properties while destroying interregional phase relationships (Supporting Information Figure S9). Empirical synchrony values (mean = 0.417 ± 0.140) significantly exceeded phase-randomized null values (mean = 0.211 ± 0.112; *t*(2686) = 39.44, *p* < 0.001, Cohen's *d* = 1.63). Similarly, empirical metastability values (mean = 0.176 ± 0.049) significantly exceeded null values (mean = 0.106 ± 0.053; *t*(2686) = 33.69, *p* < 0.001, Cohen's *d* = 1.39). These differences were highly consistent across all 14 functional networks examined (all *p* < 0.001; Supporting Information Figures S10 and S11), demonstrating that the metrics capture coordinated dynamics arising from interregional coupling rather than common statistical properties of the acquired time series. The large effect sizes (Cohen's *d* > 1.3 for both metrics) indicate that these differences are not only statistically significant but also practically meaningful (Cohen, 1988), confirming that synchrony and metastability reflect genuine neural coordination patterns that cannot be explained by chance or measurement noise.

**Figure 3.**
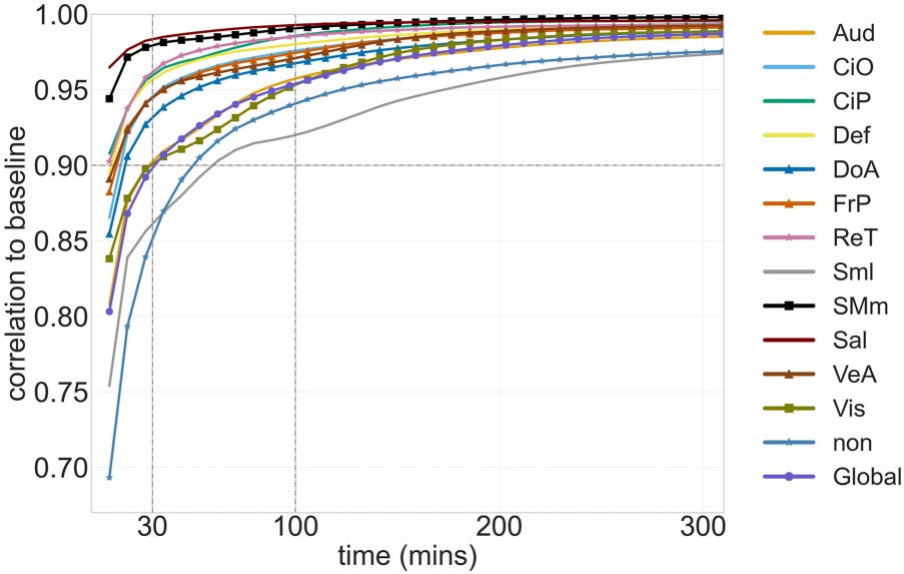
The average correlation to baseline of the static FC of each individual functional network versus the total acquisition time. Abbreviations for networks labels: Auditory (Aud), Cingulo Opercular (CiO), Cingulo Parietal (CiP), Default Mode (Def), Dorsal Attention (DoA), Fronto-Parietal (FrP), Retrosplenial (ReT), Somatomotor - lateral (Sml), Somatomotor - medial (SMm), Salience (Sal), Ventral Attention (VeA), Visual (Vis), No assignment (non), Global (whole brain).

**Figure 4.**
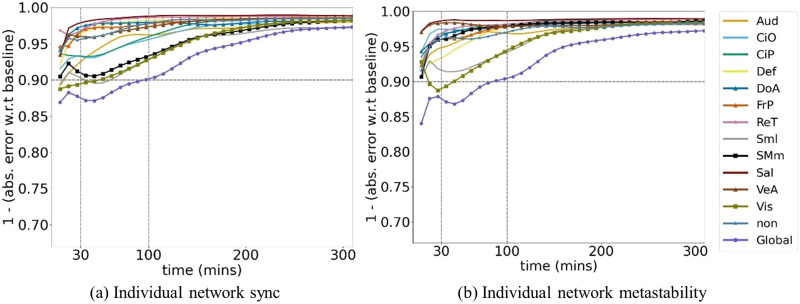
Similarity to baseline, computed as 1 minus the absolute error with respect to baseline for the scalar sync (A) and metastability (B) values of the individual functional networks versus the total acquisition time. Abbreviations for networks labels: Auditory (Aud), Cingulo Opercular (CiO), Cingulo Parietal (CiP), Default Mode (Def), Dorsal Attention (DoA), Fronto-Parietal (FrP), Retrosplenial (ReT), Somatomotor - lateral (Sml), Somatomotor - medial (SMm), Salience (Sal), Ventral Attention (VeA), Visual (Vis), No assignment (non), Global (whole brain).

We then look at the within-subject reliability of the metastability and sync values for the functional networks each taken individually. For comparison, in Figure 3, we show the correlation to baseline of the FC matrix of the individual functional networks; it can be seen that there is variability in the convergence profile across networks, but with most networks reaching a 90% similarity to baseline for 30 min of data. We observe a comparable variability across networks in the similarity to baseline profiles for the values of sync and metastability of the individual networks, as shown in Figure 4A, Supporting Information S2a, and Figure 4B, Supporting Information S2b, respectively. Notably, while FC exhibits steep declines in reliability at scan durations below 30 min, metastability demonstrates superior stability at shorter durations, maintaining higher correlations to baseline across the majority of networks examined. This suggests that metastability may be a more robust metric for studies with limited scanning time.

### The Midnight Scan Club

**Figure 5.**
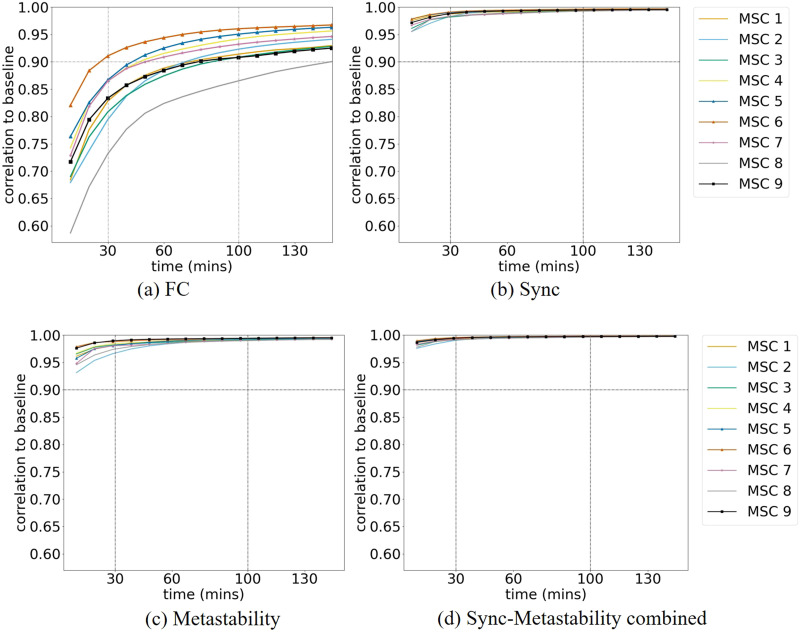
The average correlation to baseline versus the total acquisition time for the different subjects of the MSC dataset: (A) the static FC, (B) the sync feature vector, (C) the metastability feature vector, (D) the sync and metastability combined feature vector.

We replicate the HSI analysis for the data of nine subjects of the MSC dataset (Gordon, Laumann, Gilmore, et al., 2017). In Figure 5A, we can see considerable variability in the FC average correlation to baseline profile for the different subjects; for 30 min of data, the average correlation to baseline ranged from around 0.732 ± 0.048 (95% CI [0.7286, 0.7345]) to 0.911 ± 0.012 (95% CI [0.9100, 0.9114]). In comparison, Figure 5B shows a much faster convergence of average correlation to baseline for the sync feature vector using mode 0 (similarly for mode 1 in Supporting Information Figure S3a), reaching above 0.981 ± 0.009 (95% CI [0.9808, 0.9819]) average correlation at 30 min for all subjects.

The analysis of the metastability feature vector shows a slightly slower convergence than that seen for the sync vector, reaching at least 0.966 ± 0.016 (95% CI [0.9650, 0.9671]) at 30 min for all subjects (Figure 5C for mode 0 and Supporting Information Figure S3b for mode 1). When sync and metastability are combined in one feature vector, the average correlation profiles converge faster than that of the separate feature vectors, reaching an average correlation of 0.99 ± 0.006 (95% CI [0.9895, 0.9903]) for all subjects at 30 min of acquisition time (Figure 5D); Supporting Information Figure S5 shows a zoomed-in view on Figures 5B–D.

### Psilocybin

**Figure 6.**
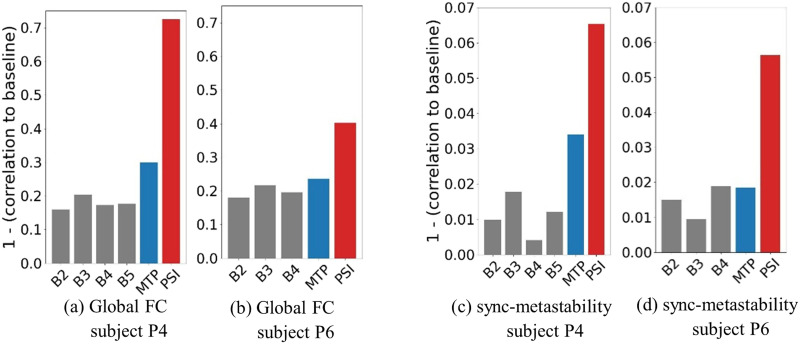
Departure from baseline for the different sessions, computed as 1 minus correlation to first baseline session, for static FC and the sync-metastability feature vector for subjects P4 (A, C) and P6 (B, D), respectively; B2 to B5 refers to baseline sessions 2–5, MTP and PSI refer to the methylphenidate and psilocybin sessions, respectively. *y*-Axes scaled independently for visual clarity.

We analyzed a subset of the data presented in Siegel et al. (2024), particularly the data for subjects P4 and P6, which exhibited the largest change in whole-brain entropy as reported in Figure 3B in Siegel et al. (2024); we use only 15 consecutive minutes from each of the available sessions. The idea is to probe the reliability of the different measures to serve as a biomarker of brain state, which is predicted by the preceding analysis to be higher for the sync-metastability than for FC, especially with limited amounts of data (less than 30 min). Indeed, we observe that the psilocybin effect manifests as a large noticeable departure from baseline as seen in Figure 6C and Figure 6D, which show 1 minus the correlation between the sync-metastability vectors of a given session and that of the first baseline session, for a given subject.

Importantly, while both FC and sync-metastability show increased departure from baseline under drug conditions, the absolute magnitude of these departures differs substantially. The sync-metastability metric exhibits departures approximately an order of magnitude smaller than FC (e.g., ~0.06 vs. ~0.6 under psilocybin, ~0.03 vs. ~0.3 under methylphenidate), noting that the *y*-axes are scaled separately for visual clarity. This suggests that sync-metastability may be a more stable metric that is less susceptible to state-related fluctuations, while still capturing meaningful differences between baseline and drug-altered states.

**Figure 7.**
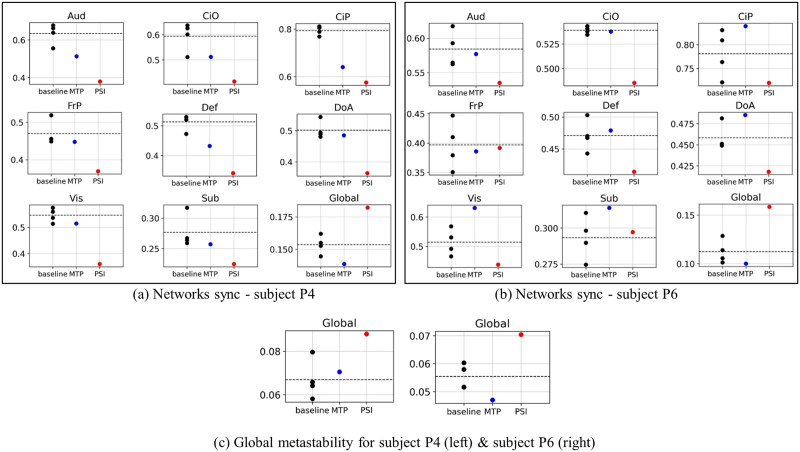
(A and B): Sync values for a subset of the individual functional networks for subject P4 and P6, respectively, for the different sessions: baseline (gray), methylphenidate (blue), and psilocybin (red). (C) Global metastability values for the different sessions: P4 (left) and P6 (right). Abbreviations for networks labels: Auditory (Aud), Cingulo Opercular (CiO), Cingulo Parietal (CiP), Fronto-Parietal (FrP), Default Mode (Def), Dorsal Attention (DoA), Visual (Vis), Subcortical (Sub), Global (whole brain).

Figure 7A and Figure 7B show the corresponding changes in the sync values for a subset of the individual networks sync, and Figure 7C shows the corresponding values for global metastability values, for subjects P4 and P6, respectively. We can see that for subject P4, there is a clear drop in sync in the majority of the networks and an increase in global metastability. The same can be said for subject P6, even though the drop in sync within individual networks is less pronounced than those seen for subject P4. For comprehensiveness, we provide the sync and metastability values for the complete set of networks in the Supplementary Materials (Supporting Information Figures S12–S15).

Figure 6A and Figure 6B show the departure from baseline graphs for the static FC matrices, which, as expected, also exhibit a drastic departure from baseline in the psilocybin session. It can be seen that P4 exhibits a smaller departure from baseline accompanied by a smaller drop in sync values observed on the individual network level.

Notably, while both FC and sync-metastability capture the drastic departure from baseline under the effect of psilocybin, the magnitude of the within-subject variability, across the baseline sessions, for the latter is an order of magnitude smaller than that seen for the former. These results are consistent with what is predicted based on the sync-metastability within-subject variability results from the HSI and the MSC datasets presented in the previous sections.

## DISCUSSION

In this work, we have attempted to systematically quantify the within-subject variability of a standard proxy of metastability and have found the metric to be at least as reliable as standard static FC, both when computed on a global whole brain level but also on a resting-state functional network level. Both metastability and FC exhibited convergence to baseline profiles as a function of acquisition time that varied across the different functional networks, with most but not all networks reaching a 0.9 correlation to baseline with 30 min of data. We thus propose combining the scalar metastability values from all the networks into one novel feature vector, which we found to exhibit an order of magnitude improvement in within-subject reliability.

Scalar metrics of metastability of BOLD fMRI, such as the standard deviation of phase synchrony that we examined here, have been employed in numerous works for looking at group differences or significant correlations between metastability and relevant behavioral or cognitive correlates. For example, in Hellyer et al. (2015), reduced metastability was observed in cases of traumatic brain injury leading to reduced cognitive flexibility and information processing; in Lee et al. (2018), metastability was found to be predictive of clinical symptoms in schizophrenia, and in Lee et al. (2019), associations between normative ranges for metastability values and several behavioral and health correlates were examined. Such studies offer valuable insight into the potential significance of measures of metastability for the understanding of brain dynamics. However, based on the analysis of the unique HSI dataset (Laumann et al., 2015; Poldrack et al., 2015), which provides 14 hr of data for the same subject, we find that when taken alone as a scalar value, global metastability exhibits an average absolute error of approximately 12% when computed for 30 min of data, which improves to 10% for 100 min of data (Figure 4B). When computed for individual functional networks, the absolute error with respect to baseline varies across the networks and ranges from a low of approximately 11% to a high of 2%. These findings reveal that metastability demonstrates superior reliability at shorter scan durations, which is an important practical distinction between FC and metastability metrics. While FC correlations decline steeply below 30 min, metastability maintains higher stability across most networks examined. This has significant implications for clinical and developmental studies where long scan durations are often impractical due to participant burden, motion artifacts, or cost constraints. For pediatric populations, patient groups with limited tolerance for scanning, or large-scale studies requiring efficient protocols, metastability may offer a more reliable metric for characterizing network dynamics. This enhanced stability at shorter durations could enable researchers to obtain meaningful measurements of temporal network variability in settings where extended acquisitions are not feasible, potentially expanding the applicability of dynamic network analyses to populations and contexts previously considered challenging for such investigations.

Interestingly, when metastability values of the individual networks are combined together as components of a 14-dimensional feature vector, the similarity to baseline is greatly enhanced, with a correlation of approximately 0.98 for 30 min of data. The latter is improved even further, to an average correlation to baseline of approximately 0.99 for 30 min of data, when the values of metastability are combined with those of the mean phase synchrony into a 28-dimensional sync-metastability feature vector.

The higher within-subject reliability observed for the combined sync-metastability feature vector likely reflects the distributed nature of coordination dynamics across functional networks. Theoretical accounts from the TCD and large-scale brain network studies propose that metastability arises from whole-brain interactions balancing segregation and integration across multiple spatial scales (Deco & Kringelbach, 2016; Kelso, 1999; Tognoli & Kelso, 2014). Whereas individual network metrics primarily capture local, mesoscopic manifestations of this balance, the combined feature vector integrates information across networks, thereby reflecting the system-level, macroscopic coordination dynamics underlying metastability. This aggregation can thus be hypothesized to capture a more stable representation of the individual's dynamic functional organization as manifesting on a whole-brain level. Moreover, the enhanced reliability of the combined sync-metastability metric can be understood through the complementary information these measures capture about underlying neural dynamics; synchrony quantifies the degree of phase alignment between regions at each moment in time, reflecting the instantaneous coordination of neural activity. Metastability, in contrast, measures the temporal variability of this coordination, capturing how flexibly networks transition between synchronized and desynchronized states. These two dimensions are theoretically orthogonal: Networks can exhibit high synchrony with either low variability (stable coordination) or high variability (flexible coordination), and conversely, low synchrony can be either stable or variable. By combining these measures into a joint feature vector, we capture both the average coordination strength and the temporal dynamics of coordination for each network. This multidimensional characterization appears to provide a more stable “fingerprint” of network behavior than either measure alone or traditional static FC. The improved reliability likely reflects the fact that while moment-to-moment fluctuations may vary across sessions, the joint distribution of coordination strength and temporal variability represents a more robust property of network organization. This framework aligns with the emerging perspective that brain network dynamics reflect a balance between integration (synchrony) and segregation (desynchronization), with healthy function requiring flexible transitions between these states. Results from the MSC (Gordon, Laumann, Gilmore, et al., 2017) dataset provide a confirmation of the robustness of the sync-metastability feature vector; while inter-subject variability exists in the correlation to baseline convergence profile, the observed average correlation for 30 min of data is approximately 0.99 for all subjects. This suggests a robust within-subject reliability, especially when compared with that observed for the same subjects for the static FC, which is seen to exhibit a considerably smaller average correlation to baseline at 30 min but also at 100 min of data.

While the improvement in reliability from an average correlation to baseline of *r* > 0.95 at ~30 min to *r* > 0.99 at longer durations may appear modest, this distinction has practical implications for precision neuroimaging and studies examining subtle individual differences or clinical effects. Based on the coefficient of determination (*r*^2^), this improvement represents a reduction in unexplained variance from ~10% to ~2%, which can be critical when true effect sizes are small, such as in longitudinal studies tracking developmental changes or interventions. Moreover, our findings reveal that reliability requirements are not uniform across networks; some achieve high stability with modest data quantities, while others require substantially longer acquisitions. These network-specific benchmarks enable researchers to make informed trade-offs in study design, selecting the minimum data quantity needed to achieve adequate reliability for their networks of interest rather than applying a one-size-fits-all approach. For studies targeting networks that stabilize quickly, shorter and more practical scanning protocols may suffice, whereas research focused on networks requiring longer acquisitions can weigh whether the additional scan time is justified for their specific research questions.

The analysis of the two subjects' data from Siegel et al. (2024) provides further validation of the strong within-subject reliability of the sync-metastability vector; it is seen that the correlation of that vector from a given baseline session to that of the first baseline session is consistent with what is suggested by the average correlation to baseline convergence profiles obtained using the HSI and MSC datasets. The sync-metastability feature vector exhibits a large departure from baseline under the effect of psilocybin, but not under methylphenidate, that is, in comparison with the magnitude of the intrinsic within-subject variability observed in the HSI and MSC datasets. Here, the HSI and MSC analysis provides a first quantification of typical correlation values expected to result from intrinsic within-subject variability for the sync-metastability feature vector, specifically no less than 0.97, as a conservative estimate. Thus, correlations to baseline values of 0.94 and 0.935, observed under the effect of psilocybin, can be interpreted as a significant departure from baseline that is not explained by intrinsic within-subject variability, a result that is consistent with what we expect in the case of a strong mind-altering substance such as psilocybin. While the magnitude of the departure from baseline for the sync-metastability feature vector is found to be much smaller than that seen for the static FC, the latter exhibits a much larger baseline-to-baseline variability when compared with the former.

It is worth noting that the analysis in Siegel et al. (2024) was done on the voxel level, whereas here, we are looking at the dynamics on a coarse-grained spatial scale, at the within-network parcels level; we see that the increase in complexity seen at the voxel and global level in Siegel et al. (2024) is reflected in our analysis as a drop in the mean phase synchrony within the individual networks that is also accompanied by an increase in global metastability; while our analysis here was limited to two sample subjects, it raises an interesting question worth addressing in future work on the relationship between synchrony measures observed at different spatial resolutions. In other words, the sensitivity of metastability and phase synchrony measures to the choice of spatial scale at which the dynamics is observed, while beyond the scope of work here is one of several open questions that follows naturally and warrants an investigation of its own. Other pertinent questions that are yet to be addressed concern the method of parcellation used for coarse graining the signals from the voxel to the parcel level. While we have used the Gordon parcellation (Gordon et al., 2016; Gordon, Laumann, Adeyemo, & Petersen, 2017), it would be interesting to compare the within-subject reliability convergence profiles obtained here to those that would result using different anatomical atlas-based parcellations (Lawrence et al., 2021). The choice between anatomical and functional brain parcellations is potentially consequential for analyzing phase synchrony and metastability in resting-state fMRI networks. Anatomical atlases define regions based on structural landmarks that do not necessarily respect functional boundaries, leading to signal averaging across functionally heterogeneous voxels within each region. This can blur temporal dynamics, dampen phase coherence fluctuations, and possibly reduce measured metastability by mixing signals from functionally distinct neural populations. In contrast, functional atlases like the Gordon parcellation define regions based on more coherent functional activity patterns, with the aim of designating functionally homogeneous parcels that better represent distinct neural populations with coherent dynamics. This delineation of functional boundaries likely provides a more accurate detection of phase synchrony fluctuations and a better characterization of metastable state transitions, that is, given that the parcellation is presumed to align with the underlying functional architecture where these dynamics naturally emerge. While a thorough characterization of how different parcellation schemes affect measures of network metastability and phase synchrony is beyond the scope of the present work, this remains an important question for future investigation.

In addition, another issue worth exploring is the effect of the use of alternative functional network assignment methods for mapping voxels or parcels onto the different resting-state networks. Predefined functional networks impose a fixed group-average template on what is actually a highly individual and context-dependent organization of brain function. Functional network topography could vary substantially across individuals, and applying one template to all participants ignores meaningful individual differences that may be behaviorally or clinically relevant. Moreover, predefined networks typically follow a “hard assignment” of each region to a single network, failing to capture the flexible reconfiguration of functional communities that could occur as regions participate in multiple networks over time. Alternative approaches such as data-driven community detection using independent component analysis individual-specific parcellations, or multi-layer network assignments may better capture the true complexity of functional brain organization, though these methods present their own methodological challenges, specifically in cross-study comparability.

A limitation of the current study is the exclusion of the cerebellum, known to be implicated in psychiatric disorders. The Gordon parcellation used here was designed primarily for cortical surface analysis using gradient-based parcellation techniques and does not adequately cover cerebellar structures. Specifically, the cerebellum may be captured incompletely depending on the acquisition sequence, and deep cerebellar nuclei are small relative to typical fMRI spatial resolution, often occupying only a few voxels. Moreover, adequately characterizing the cerebellum's contribution to network metastability would require a refined cerebellar parcellation scheme that respects its fine-scale functional organization and unique latticed circuit architecture. Future investigations examining metastability in cerebellar-cortical circuits using appropriate cerebellar parcellations would represent an important avenue for understanding the neurobiological mechanisms underlying psychiatric disorders, particularly in schizophrenia where cerebellar dysfunction has been well-documented.

Finally, while we have used EMD to extract the narrow band components from which the phase signals were extracted, and have thus avoided the need to prescribe the exact frequency ranges for the modes, future work could possibly look at the sensitivity of the results to, instead, using a prespecified frequency range to bandpass the signals, as well as the effect of the exact choice of cutoffs for that frequency range, which takes on several common values in the literature.

Our work here has aimed to provide a first systematic assessment of the within-subject variability of metastability in order to quantify the confidence with which we can interpret the dependence of variability in metastability on group effects or other factors. Having examined reliability as a function of duration of acquisition, our results suggest that our proposed within-network sync-metastability feature vector can serve as a reliable dynamics-based brain state metric for use in future computational modeling or clinical studies. Our study focused primarily on establishing within-subject reliability requirements for synchrony and metastability metrics, with the HSI dataset providing exceptional temporal depth in a single individual and the MSC dataset demonstrating generalizability across nine subjects. However, several limitations should be noted. First, the modest sample sizes, particularly in the MSC (*n* = 9) and psilocybin (*n* = 2) datasets, limit conclusions about population-level generalizability. Intersubject variability in healthy populations, and especially the heterogeneity characteristic of clinical populations, represents an additional layer of complexity beyond the within-subject reliability we have characterized. Our findings establish baseline requirements for obtaining stable individual measurements, a necessary foundation for population studies, but adequately powered investigations of clinical or developmental populations will require both sufficient within-subject data, as benchmarked here, and sufficient sample sizes to capture between-subject variability. Second, network-specific reliability characteristics observed in healthy adults may differ in clinical populations where underlying pathophysiology could alter the temporal dynamics of network organization. Future work should examine whether the data quantity requirements we identify generalize to patient populations or require adjustment based on disease-specific patterns of network variability.

Our findings bring forth the challenging question of establishing universal clinical significance thresholds for reliability metrics as the required level of measurement precision depends on the specific research question and expected effect sizes. However, we can offer general guidance for future applications. For studies examining large effects, for example, gross differences between patient and control groups, correlation to baseline values of *r* > 0.90 (*r*^2^ > 0.81, ~19% unexplained variance) may be sufficient, typically achievable with 20–40 min of data for most networks. For studies targeting moderate effects, for example, treatment responses, developmental changes, *r* > 0.95 (*r*^2^ > 0.90, ~10% unexplained variance) is advisable, generally requiring 30–60 min depending on the network. For precision medicine applications aiming to characterize individual differences or predict outcomes, higher thresholds of *r* > 0.97–0.99 (*r*^2^ > 0.94–0.98, < 6% unexplained variance) may be necessary, potentially requiring 60–150+ min of data. Importantly, these are within-subject reliability estimates; adequately powered clinical studies must also consider sample size requirements to detect between-group or between-subject effects. Researchers should consider both the networks of interest, given the substantial variability we observe in data requirements across networks, and the expected effect size when designing acquisition protocols. Pilot studies assessing reliability in the specific population of interest are recommended, as clinical populations may exhibit different reliability characteristics than the healthy adults examined here.

Ultimately, the work is also intended to pave the way for future studies that can fill the gap in our knowledge regarding the sensitivity of dynamic FC measures in general, and metastability in particular, to the heterogeneity in pertinent signal acquisition and postprocessing factors. Future work could leverage this reliable feature vector in supervised learning frameworks to distinguish patients from controls in out-of-sample classification analyses or in unsupervised approaches to identify novel disease subtypes based on dynamics-based phenotypes, potentially advancing precision psychiatry applications. Additionally, sync-metastability measures could serve as target engagement biomarkers for evaluating the effects of neuromodulatory brain stimulation interventions targeting precision functional networks in neurological or psychiatric disorders (Nahas et al., 2025).

## METHODS

### Data Acquisition and Processing

#### HSI dataset.

The details of data acquisition and processing for the HSI (MyConnectome) dataset have been previously described (Laumann et al., 2015; Poldrack et al., 2015); the data were provided by the latter authors and is available at openneuro.org (https://openneuro.org/datasets/ds000031/versions/2.0.2). Briefly, in this dataset, a single participant (primary subject from Laumann et al., 2015), a healthy adult male, underwent scanning sessions approximately twice weekly over the course of 532 days, accumulating 84 usable 10-min resting-state fMRI runs. Data were acquired using a 3 T Siemens Skyra scanner at the University of Texas at Austin. T1-weighted structural images were acquired with: TE = 2.14 ms, TR = 2500 ms, flip angle = 7°, 1-mm isotropic voxels. BOLD fMRI data were acquired with: TE = 30 ms, TR = 1.16 s, flip angle = 63°, 2.4-mm isotropic voxels, 60 oblique axial slices acquired with multiband acceleration factor = 4. Motion censoring was not applied to the HSI data in our analysis. This decision was based on the exceptionally low motion characteristics of this dataset: Laumann et al. (2015) reported that motion censoring at framewise displacement (FD) > 0.5 mm resulted in 93% ± 9% frame retention, indicating minimal head motion. To maintain consistent session lengths for our concatenation-based sampling approach and given the high baseline data quality, we used all available frames. The small proportion of frames with FD > 0.5 mm (< 7% on average) was judged to have negligible impact on reliability estimates computed from extensive temporal averaging across 84 sessions.

#### MSC dataset.

The MSC dataset consists of 10 healthy young adults (5 female, mean age = 29.3 years, age range = 24–34 years) who were members of the research team at Washington University. Anonymized raw signals were obtained from the openneuro.org open-source database: https://openneuro.org/datasets/ds000224/versions/1.0.4). The MSC participant's 4 T1w (sagittal 224 slices 0.8 mm × 0.8 mm × 0.8 mm, TE = 3.74 ms, TR = 2400 ms), 4 T2w (sagittal 224 slices, 0.8 mm × 0.8 mm × 0.8 mm, TE = 479 ms, TR = 3200 ms), BOLD data (10 days, 30 min each resting state, voxel size = 4 mm × 4 mm × 4 mm; 36 slices, TR = 2.2 s, TE = 2.7 ms), and gradient echo field map MRIs (Siemens TRIO 3 T MRI) were then processed with the publicly available ABCD-BIDS (Adolescent Brain Cognitive Development study, using the Brain Imaging Data Structure) pipeline, which is a modified version of the HCP (Human Connectome Project) Pipeline (https://github.com/DCAN-Labs/abcd-hcp-pipeline), which uses the cifti file format (Glasser et al., 2013). All sessions for each subject were concatenated across collection days to ensure that the BOLD data were projected onto the same cortical ribbon surface mesh. Task and resting-state fMRI data were processed together; however, only resting-state data were used for further analysis. After the volumetric time series is projected onto the cortical surface, DCAN-BOLDS (Developmental Cognitive and Neuroimaging Lab-Blood Oxygenation Level Dependent Signals) Processing was used to perform nuisance regression on dense time series data. In addition, motion censoring is performed according to Power et al. (2014), using a framewise displacement threshold of 0.2 mm. The motion numbers produced in the FMRI Volume step of the pipeline were filtered to remove respiratory motion artifacts. In addition, using the surviving frames, the standard deviation of the bold was calculated across all grayordinates, and frames that had excessively high standard deviations were also removed from the time series. Dense time series were parcellated using the Gordon Parcellation (Gordon et al., 2016) using the workbench command “cifti parcellate” using Connectome workbench v.1.5.0–2.1.0. For our main MSC analysis, we used the preprocessed data output from the DCAN-BOLDS pipeline, which included nuisance regression and removal of volumes with excessively high signal variance but did not apply framewise motion censoring based on framewise displacement thresholds. This approach maintains consistency with our HSI analysis. To verify robustness to motion censoring choices, we conducted a supplementary reanalysis applying FD > 0.2 mm censoring and restricting analysis to subjects retaining > 60 min of usable data (*n* = 6 subjects; Supporting Information Figure S6), which yielded qualitatively consistent results.

#### Psilocybin dataset.

The Psilocybin dataset DICOM (Digital Imaging and Communications in Medicine) files were shared by Siegel and colleagues (Siegel et al., 2024) and can be found in BIDS (Brain Imaging Data Structure) format at openneuro.org (https://openneuro.org/datasets/ds006072/versions/1.0.6). Details regarding acquisition parameters and study design have been previously described (Siegel et al., 2024). Briefly, healthy adults (*n* = 7, aged 18–45 years) were scanned approximately every other day at a consistent time of day to minimize diurnal effects on FC. We here analyzed the data from only two of the subjects (designated P4 and P6 in Siegel et al., 2024); Subject P4 completed four baseline sessions, one methylphenidate session (20 mg oral), and one psilocybin session (25 mg oral). Subject P6 completed five baseline sessions, one methylphenidate session, and one psilocybin session. Both subjects were healthy adults with prior psychedelic experience (as required by the study protocol) but no psychedelic use within 6 months prior to the study. Sessions were conducted approximately every other day to allow washout between pharmacological manipulations. Neuroimaging was performed on a Siemens Prisma 3 T scanner at the Washington University Medical Center. Structural scans included T1-weighted and T2-weighted images acquired at a 0.9-mm isotropic resolution with real-time motion correction. Structural scans from different sessions were averaged together for FreeSurfer segmentation and nonlinear atlas registrations. BOLD fMRI data were acquired using a multi-echo echo-planar imaging sequence with the following parameters: 2.0-mm isotropic voxels, five echo times (TE = 14.20, 38.93, 63.66, 88.39, 113.12 ms), TR = 1.761 s, flip angle = 68°, multiband acceleration factor = 6, in-plane acceleration (GRAPPA [GeneRalized Autocalibrating Partial Parallel Acquisition]) factor = 2, and 72 axial slices (144-mm coverage). Each resting-state scan included 510 frames (duration: 15 min 49 s) plus three additional frames at the end to estimate electronic noise for thermal denoising. Two 15-min resting-state fMRI scans were acquired during each session, during which participants were instructed to hold still and fixate on a white crosshair presented on a black background. Head motion was monitored in real-time using Framewise Integrated Real-time MRI Monitoring software (FIRMM; Dosenbach et al., 2017). DICOM data were converted to BIDS format using Dcm2Bids (Boré et al., 2023), and NOise Reduction with DIstribution Corrected (NORDIC) PCA thermal denoising was applied (Vizioli et al., 2021). Data were processed using fMRIPrep Version 24.0 or greater (Esteban et al., 2019), followed by postprocessing with XCP-D (the eXtensible Connectivity Pipeline-DCAN) (Mehta et al., 2024) using a respiratory filter of 12–18 breaths per minute. Data were output in standard 91,282 grayordinate resolution. To ensure that each functional run was mapped to an identical surface mesh generated during the FreeSurfer processing step, each separate testing day was treated as a unique task. For 500 grayordinates in the inferior temporal lobe (a region with known signal dropout) where BOLD data could not be projected, values were spatially interpolated using the mean and standard deviation of grayordinates in the same hemisphere. Dense time series data were then parcellated using the Gordon parcellation (Gordon et al., 2016). Motion censoring was performed by removing time points with framewise displacement > 0.4 mm. Data retention rates after motion censoring were: Subject P4: 99% for baseline sessions, 99% for methylphenidate session, 89% for psilocybin session; Subject P6: 99% for baseline sessions, 99% for methylphenidate session, 97% for psilocybin session.

### Computing Sync and Metastability

The first step for computing metastability is the bandpass filtering of the postprocessed signals into narrow band components from which the phase signals can be extracted using the Hilbert transform. One commonly used choice of a filtering frequency band is the 0.04–0.07 Hz (Glerean et al., 2012), but several other choices have also been used in different applications. For the sake of comprehensiveness, we will refrain from making such an a priori choice here, and instead, we will use the EMD method (Huang et al., 1998; Liang et al., 2005) to extract the narrow band components of the BOLD fMRI signals. EMD is an iterative numerical method that empirically decomposes a signal into the sum of its intrinsic mode functions (IMFs), which are successively slower narrow band components. This allows us to study the metastability of the intrinsic oscillatory dynamics that could potentially be at play at different timescales; the two leading resulting IMFs are then the oscillatory components within the 0.04- to 0.07-Hz and 0.01- to 0.04-Hz frequency bands, which we refer to as “mode 0” and “mode 1,” respectively; not surprisingly, mode 0 corresponds closely to the commonly proposed functionally relevant frequency band (Glerean et al., 2012). The EMD computation was done using a custom code utilizing the “*emd*” package (Quinn et al., 2021) in *Python 3.11*, and the corresponding phase signals were extracted using the “*emd.spectra.frequency_transform*” function, which implements the Hilbert transform method.

**Figure 8.**
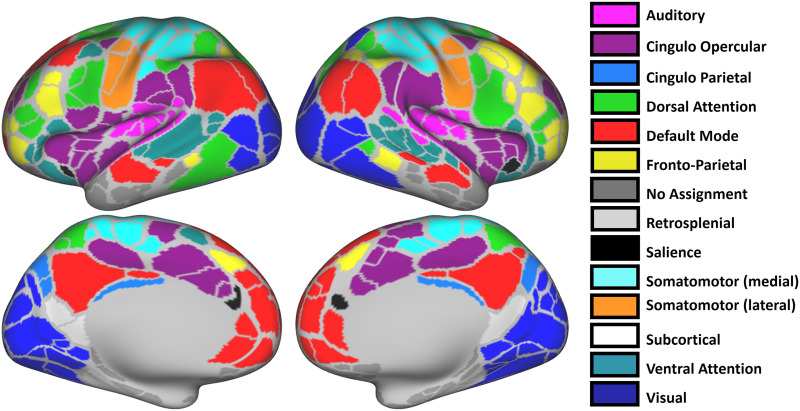
The topography of the functional networks according to the Gordon parcellation.

Once we have the instantaneous phase signals, *θ_p_*(*t*), for the individual parcels in a given functional network, *n*, the instantaneous phase synchrony for that network is given by the Kuramoto order parameter defined as *φ_n_*(*t*) = |〈*e*^*iθ_p_*(*t*)^〉_*p*∈*n*_|; “sync” and “metastability” are then taken to be the mean and standard deviation values of *φ_n_*(*t*) over the duration of a given acquisition period (Shanahan, 2010). This is done for each of the resting-state functional networks depicted in Figure 8 (see Gordon, Laumann, Adeyemo, & Petersen, 2017, for full details on the network assignment method); we exclude the subcortical network in the analysis of the HSI data as it was not available in that dataset, and we add a “Global” network that consists of all 333 parcels, giving a total of 14 networks whose sync and metastability values are then used as the elements of the sync-metastability feature vector. For the MSC and the psilocybin datasets, the subcortical regions time series were available and consisted of 19 additional parcels, leading to a total of 352 parcels and a corresponding total of 15 networks.

### Computing the Within-Subject Variability

Similar to the approach followed in Laumann et al. (2015), for the highly sampled brain dataset, 84 sessions were repeatedly split into two randomly selected subsets of 42 sessions; we perform 1,000 unique random iterations. The phase time series for each parcel is computed for the individual 10-min sessions and then concatenated across sessions before computing the sync and metastability values; the “baseline” values are computed using concatenated phase time courses from all the sessions of one subset (*n* = 42; ∼370 min of data); we discard the first minute of each session as was done in Laumann et al. (2015) to exclude transient fMRI responses evoked by the scan start and noise-cancelling headphones; and additional 5 data points (5.8 s) were discarded from the beginning and end of each phase signal to avoid edge artifacts that might arise from the EMD method, resulting in a net 8.8-min retained data per session. The similarity between the “baseline” metric vector and that derived from varying amounts of the remaining subset of sessions was computed using Pearson's correlation; we then compute the average and standard deviation, across the 1,000 random iterations, for this correlation value, as computed at increasing amount of data acquisition duration, ranging from 8.8 to 370 min, with increments of 8.8 min.

More specifically, from the second subset of 42 sessions, we incrementally sampled increasing amounts of data, ranging from 8.8 to 370 min in 8.8-min increments. For example, to obtain 8.8 min of data, we randomly selected one complete session; for 2 × 8.8 min, we selected two sessions and so on. At each duration increment, we computed the similarity (Pearson correlation for network vectors) between the metrics derived from this subset and those from the baseline. This process was repeated across 1,000 unique random iterations, each with a different random split of the 84 sessions into two subsets of 42 sessions. We then calculated the mean and standard deviation of the similarity metric across these 1,000 iterations at each data duration level. This approach is conceptually similar to bootstrap resampling but uses random split-half partitioning without replacement rather than sampling with replacement, allowing us to quantify the stability and reliability of our metrics as a function of data quantity.

The same sampling process was applied for computing the within-subject variability profiles for static FC. At every iteration, for the baseline and sample data of a given duration, FC was computed as the Fisher *z*-transformed pairwise correlation matrix between the brain parcels time series, in line with what was done in Laumann et al. (2015).

It is not feasible to compute the correlation when looking at the within-subject variability of the individual scalar values of sync and metastability of the functional networks, as opposed to the sync-metastability vector; we instead compute the absolute error with respect to baseline over the 1,000 random iterations for a given network and then plot 1 minus that value to depict the level of similarity to baseline. Our choice of similarity metric here depends on the dimensionality of the data being compared. When assessing the reliability of the joint pattern across all networks, that is, comparing vectors containing the synchrony or metastability values for all 14 networks, we use Pearson correlation between the baseline vector and test vector. This captures whether the relative relationships among networks remain stable. When assessing the reliability of individual network metrics, we are comparing scalar values for a single network and correlation is undefined for single numbers, that is, we want to quantify the similarity between two scalars for a given sampling iteration. Thus, we compute the absolute error between the test value and baseline value, and then calculate 1 − (absolute error/ baseline value) to express similarity on a scale comparable to correlation, where values closer to 1 indicate better agreement with baseline. Both approaches quantify convergence to baseline but are appropriate for different data structures, the correlation for multivariate patterns and the normalized absolute error for univariate values.

We note that our analyses examine reliability across 14 functional networks without formal multiple comparison correction. The networks are not expected to be statistically independent, as they make up one complex high dimensional system; our analyses characterize data quantity requirements for reliable within-network measurements rather than test statistical hypotheses about between-network differences. We interpret the variability across networks as observed heterogeneity in data requirements rather than make formal statistical claims about which networks differ significantly from others.

The same approach for computing within-subject variability was applied to the data of each of the individual subjects in the MSC; we leave out subject 10 as it had missing data for two of its sessions. For each subject, there are 10 sessions of 30 min of data each; we divide each of the sessions into three shorter sessions of 10 min each so that we end up with 30 sessions of 10 min each for a given subject. The 30 sessions were repeatedly split into two randomly selected subsets of 15 sessions, and 1,000 unique random iterations were performed. The analysis is identical to that for the HSI dataset with the distinction that the baseline session has a total of 150 min of concatenated data and the correlations and similarity values are computed at increasing amounts of data acquisition duration, ranging from 10 to 150 min.

To assess whether synchrony and metastability metrics capture genuine neural signal versus statistical artifacts, we compared empirical metric values against phase-randomized surrogate data (Prichard & Theiler, 1994; Theiler et al., 1992). For each of the 84 sessions, we generated 100 phase-randomized surrogates by computing the Fourier transform of each brain parcel time series, randomizing the phase components independently for each parcel while preserving the amplitude spectrum, and then applying the inverse Fourier transform. This procedure destroys coordinated phase relationships between regions while preserving the temporal autocorrelation structure and power spectrum of each individual time series. We computed synchrony and metastability metrics for one randomly selected surrogate from each session (yielding 84 null samples matched to the 84 empirical sessions) and compared empirical versus null distributions using independent *t* tests and Mann–Whitney *U* tests. Effect sizes were quantified using Cohen's *d*. Network-specific comparisons assessed whether differences between empirical and null data were consistent across the examined network parcellation.

All computations were performed with a custom written code using the “NumPy” and “*SciPy*” packages in *Python 3.11*.

## Acknowledgments

We would like to thank Timothy O. Laumann for sharing the HSI dataset.

## Supporting Information

Supporting information for this article is available at https://doi.org/10.1162/NETN.a.537.

## Author Contributions

Hiba Sheheitli: Conceptualization; Formal analysis; Investigation; Methodology; Software; Visualization; Writing – original draft; Writing – review & editing. Robert Hermosillo: Data curation; Software; Visualization; Writing – original draft; Writing – review & editing. Gracie Grimsrud: Data curation; Software. Thomas Madison: Data curation; Software. Oscar Miranda Dominguez: Methodology; Writing – review & editing. Steven Nelson: Conceptualization; Methodology; Writing – review & editing. Damien Fair: Conceptualization; Methodology; Writing – review & editing. Ziad Nahas: Conceptualization; Methodology; Writing – review & editing.

## Funding Information

Hiba Sheheitli, University of Minnesota MnDrive Initiative.

## Data and Code Availability

Data used are available via open sources referenced in the text. The custom code used to perform the computations is available upon request from the corresponding author.

## Supplementary Material



## TECHNICAL TERMS

Metastability: The brain's tendency to spontaneously switch between recurring coordination states, reflecting a balance between integration and segregation of neural activity.
Functional connectivity: Statistical dependencies between brain regions' activity patterns, typically measured as correlation between regional time series over entire scanning sessions.
Synchrony (phase synchrony): The degree to which brain regions align their oscillatory activity in time, measured by phase relationships between signals.
Brain parcel: A spatially contiguous group of brain voxels with similar functional properties, treated as a single unit for analyzing brain activity.
Brain functional network: A set of brain regions that show coordinated activity patterns, working together to support specific cognitive, sensory, or motor functions.
Brain parcellation: The division of the brain into distinct regions based on anatomical boundaries, functional properties, or connectivity patterns for systematic analysis.
Resting-state fMRI: Brain imaging technique measuring spontaneous neural activity while participants rest quietly without performing specific tasks, revealing intrinsic network organization.
Gordon parcellation: A brain atlas dividing cortex into 333 distinct regions based on functional connectivity patterns, organizing them into functional networks.
Within-subject reliability: The consistency of measurements obtained from the same individual across different sessions or data subsets, indicating metric stability.
Kuramoto order parameter: A mathematical measure quantifying the degree of synchronization among oscillators, ranging from 0 (complete desynchronization) to 1 (perfect synchronization).
