# Supplementary material for: Metastability of resting-state bold fMRI as a reliable biomarker of individual brain dynamics: An interrogation of within-subject variability as a function of total acquisition time
Source: Netw Neurosci. 2026 Apr 22;10(2):281–302. doi: 10.1162/NETN.a.537 (PMC13108507; doi:10.1162/NETN.a.537)
Supplement: Supplementary file 1 [file netn-10-2-281-s001.pdf]

Supplementary Material

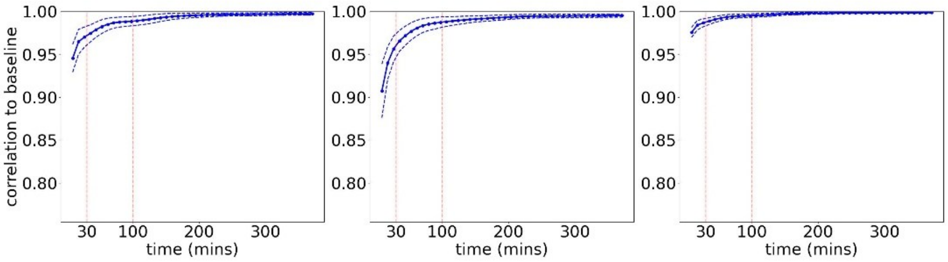

(a) Sync (b) Metastability (c) Combined

Figure S1: the average (solid line) correlation to baseline vs. the total acquisition time for the mode 1 (0.01-0.04Hz) sync feature vector (a), metastability feature vector (b), sync & metastability combined in one feature vector (c); dashed lines show ± standard deviation.

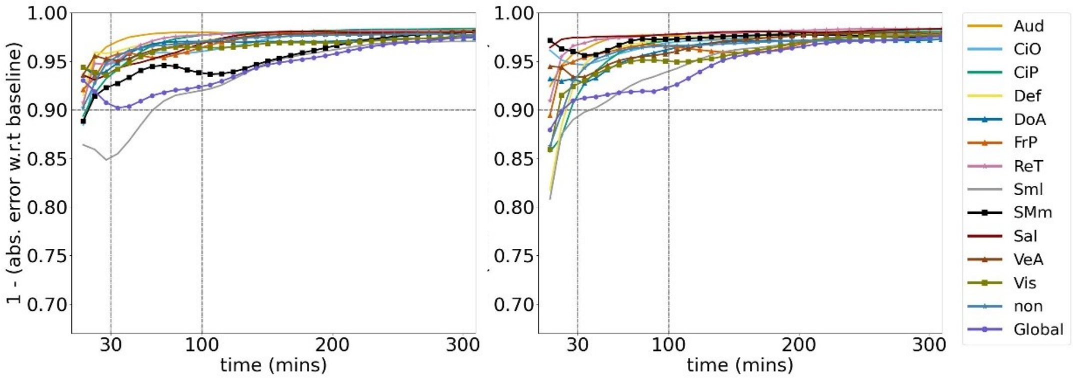

(a) Individual network sync (b) Individual network metastability

Figure S2: similarity to baseline, computed as 1 minus the absolute error with respect to baseline vs. the total acquisition time, for mode 1 scalar sync (a) and metastability (b) values for the individual functional networks.

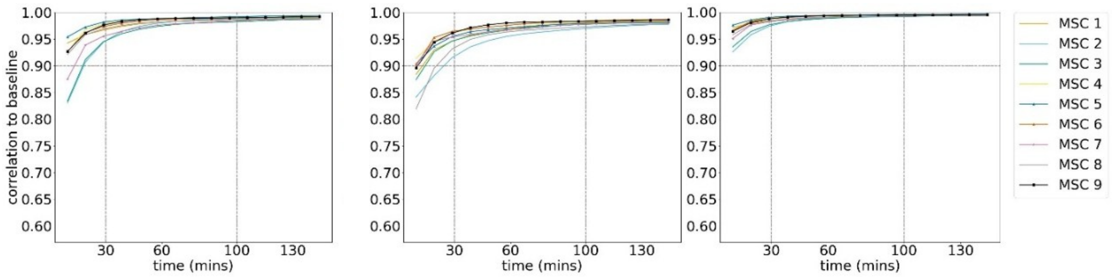

(a) (b) (c)

Figure S3: the average correlation to baseline vs. the total acquisition time for the different subjects of the MSC dataset for the mode 1 sync feature vector (a), metastability feature vector (b), sync & metastability combined feature vector (c).

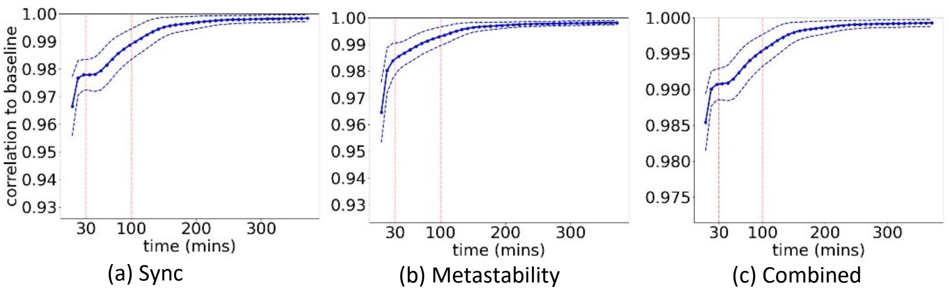

Figure S4: same as in figure 2(b), (c), (d) but with a rescaled y-axis (a zoomed-in view).

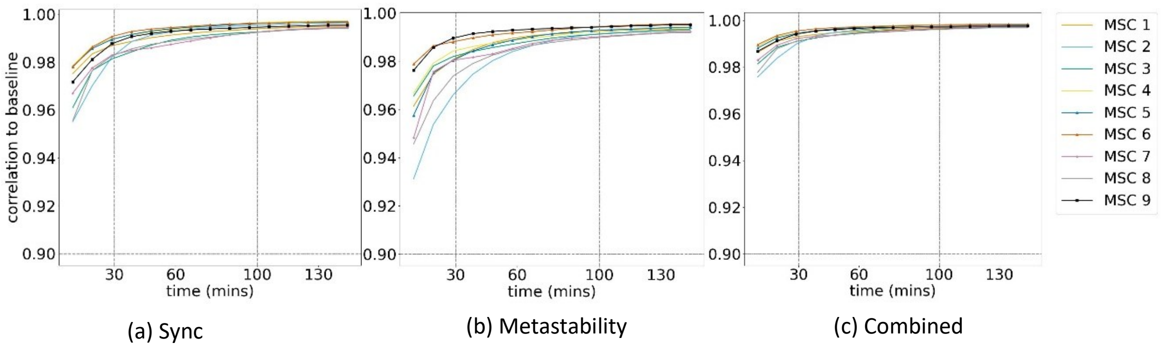

Figure S5: same as in figure 5 (b), (c), (d) but with a rescaled y-axis (a zoomed-in view).

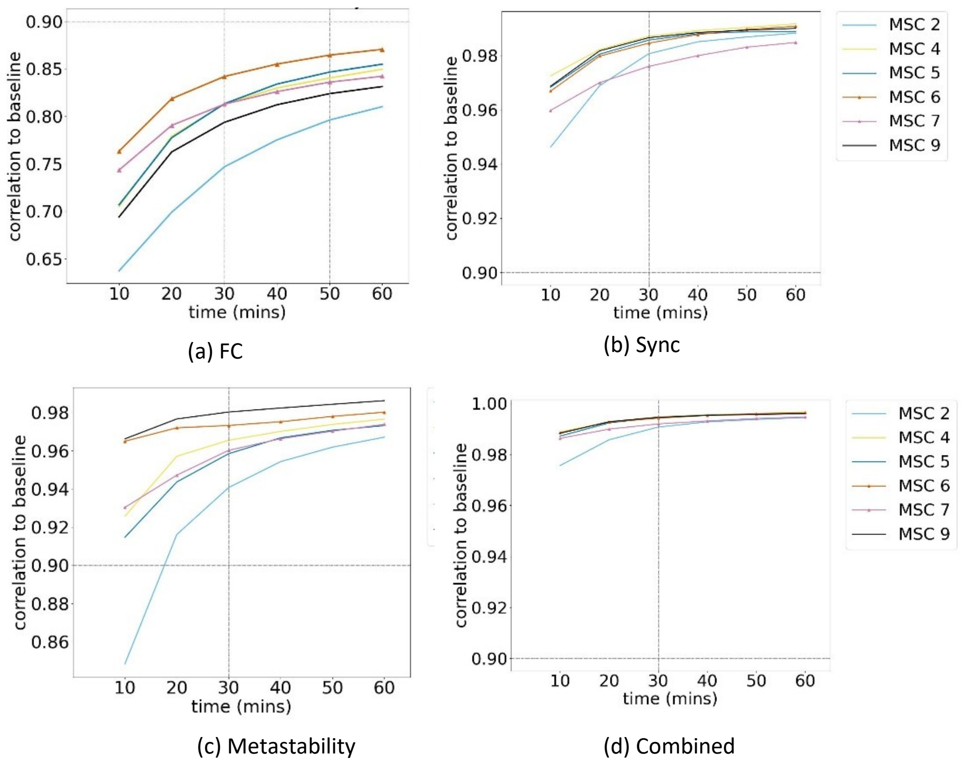

*Figure S6: the average correlation to baseline vs. the total acquisition time for the different subjects of the MSC dataset, **with motion censoring**; (a) the static FC, (b) sync feature vector, (c) metastability feature vector, (d) sync & metastability combined feature vector.*

Cross-Correlation Analysis of Network Metrics

To characterize relationships between network metrics, we computed cross-correlation matrices from baseline data. For each of the 1000 split-half iterations, synchrony and metastability values across all networks were concatenated into a 28-dimensional feature vector (14 synchrony values + 14 metastability values). Pearson correlations were computed between all pairs of features across iterations, yielding a 28×28 matrix quantifying how metrics co-vary across different data samples, presented in Figure S6 above.

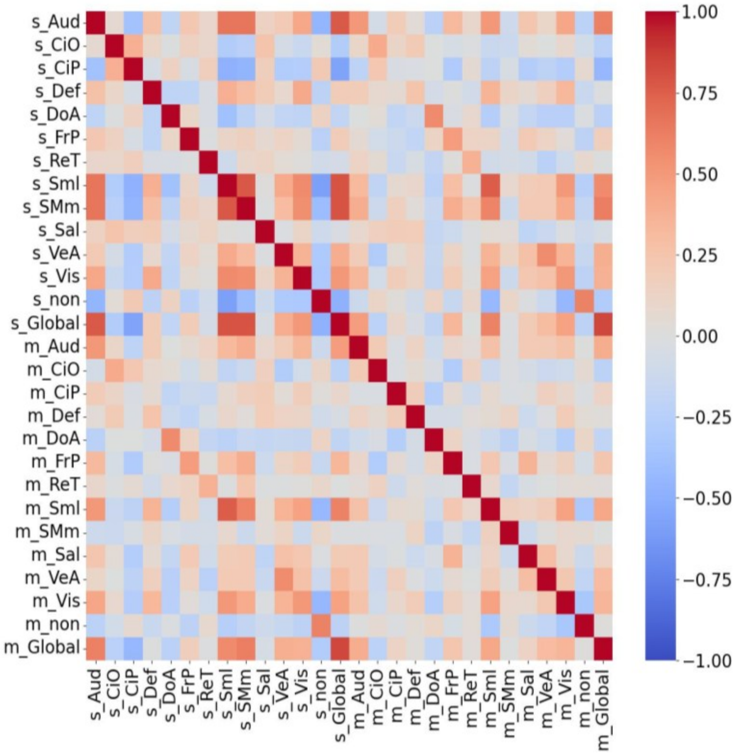

Figure S7: Cross-correlation matrix between synchrony and metastability metrics across functional networks. Heat map showing the Pearson correlation coefficients between all pairwise combinations of synchrony (s_) and metastability (m_) values across the 14 functional networks plus the global network, computed from baseline data (370 minutes, 1000 random split-half iterations) in the HSI dataset.

Analyzing The Cross-Correlation Between Metrics as a Function of Duration of Data

To assess how the relationships among network metrics stabilize with data duration, we quantified the convergence of cross-correlation across network metrics by computing the 28x28 correlation matrix, similarly to what was done for the baseline samples (shown in Figure S6). From the correlation matrices computed at each data duration, we extracted three summary statistics: (1) mean absolute correlation among all synchrony values across networks (Sync-Sync), computed as the average of the absolute values in the upper triangle of the synchrony-synchrony block (rows/columns 1-14) of the correlation matrix; (2) mean absolute correlation among all metastability values (MS-MS), computed similarly from the metastability-metastability block (rows/columns 15-28); and (3) mean absolute cross-correlation between synchrony and metastability (Sync-MS), computed as the average absolute value of all elements in the off-diagonal blocks relating synchrony of one network to metastability of others. These

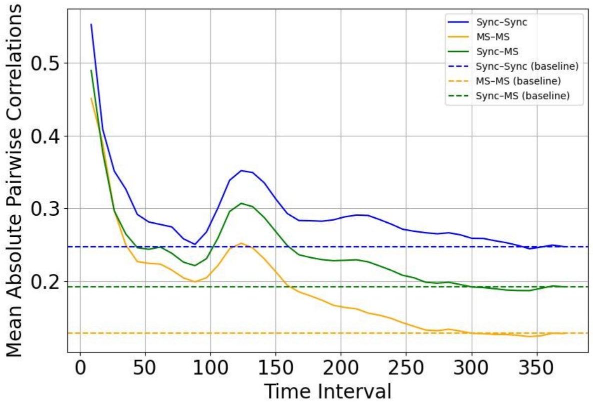

Figure S8: Convergence of cross-correlation structure with increasing data duration. Mean absolute pairwise correlations within and between synchrony and metastability metrics as a function of data acquisition duration in the HSI dataset. Blue line shows the average correlation among synchrony values across networks (Sync-Sync), orange line shows average correlation among metastability values (MS-MS), and green line shows average cross-correlation between synchrony and metastability metrics (Sync-MS). Dashed horizontal lines indicate the baseline values computed from the full 370 minutes of data.

metrics were computed at each data duration from 10 to 370 minutes and compared against baseline values computed from the full 370-minute dataset. This analysis characterizes how the multivariate covariance structure of network metrics stabilizes with increasing data acquisition.

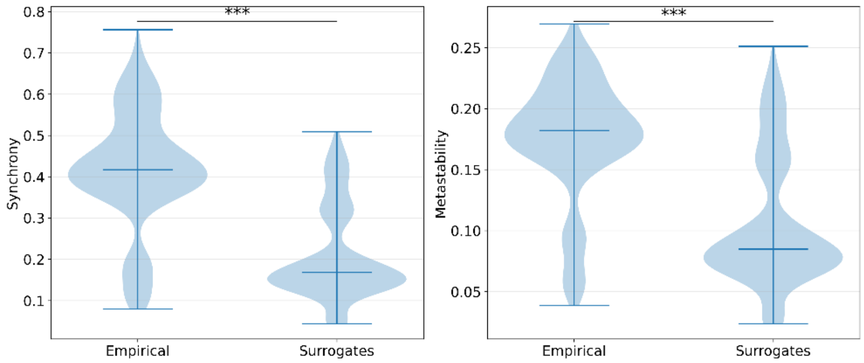

Figure S9: Violin plots comparing distributions of synchrony (left) and metastability (right) values between empirical data and phase-randomized surrogate data across all sessions and networks. Empirical values significantly exceed null for both metrics (synchrony: $p < 0.001$, Cohen's $d = 1.63$; metastability: $p < 0.001$, Cohen's $d = 1.39$).

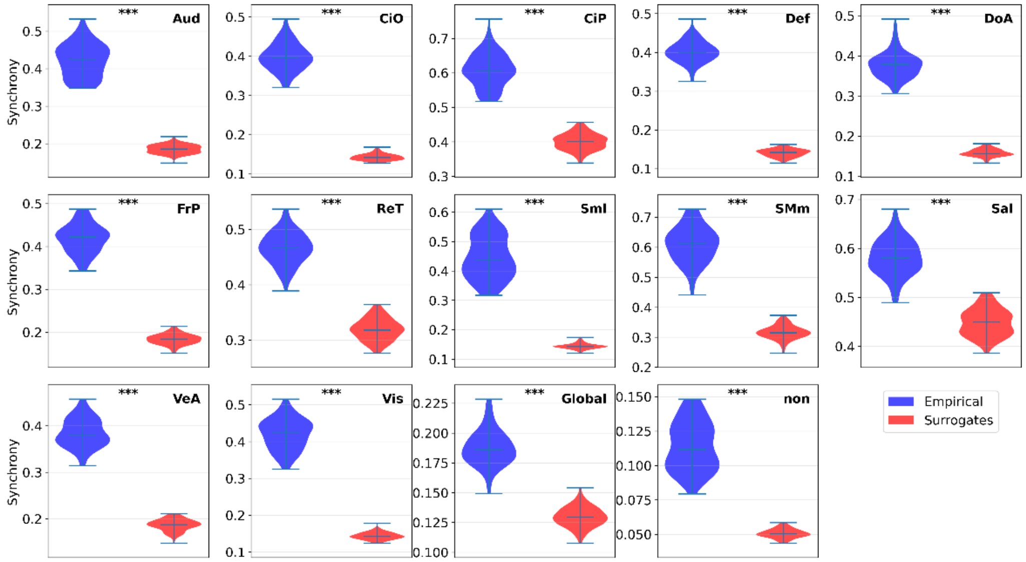

*Figure S10: Network-specific distributions for synchrony values across 14 functional networks; empirical data (blue) and phase-randomized surrogates (red); *** denotes p < 0.001.*

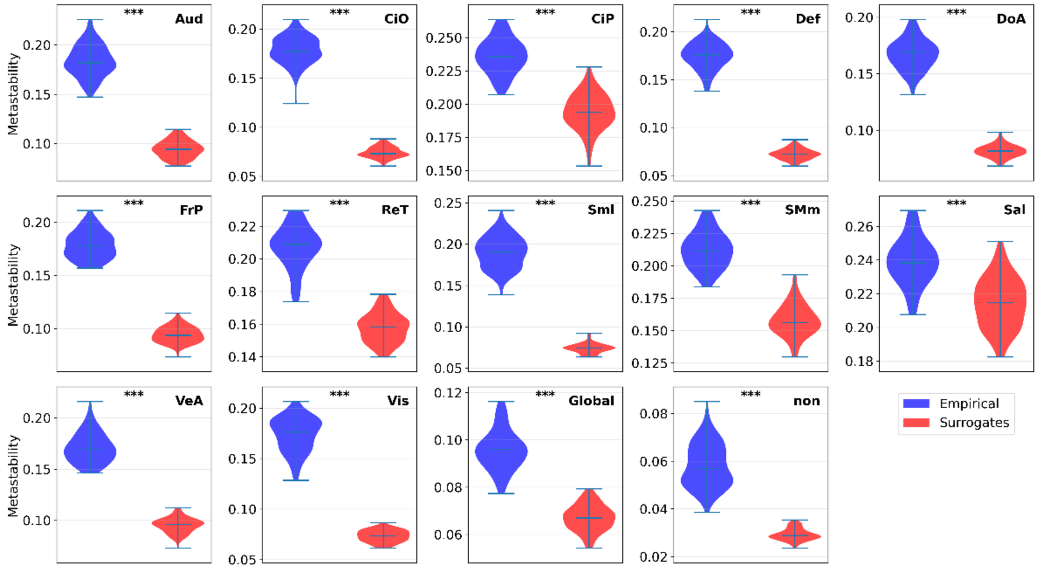

*Figure S11: Network-specific distributions for metastability values across 14 functional networks; empirical data (blue) and phase-randomized surrogates (red); *** denotes p < 0.001.*

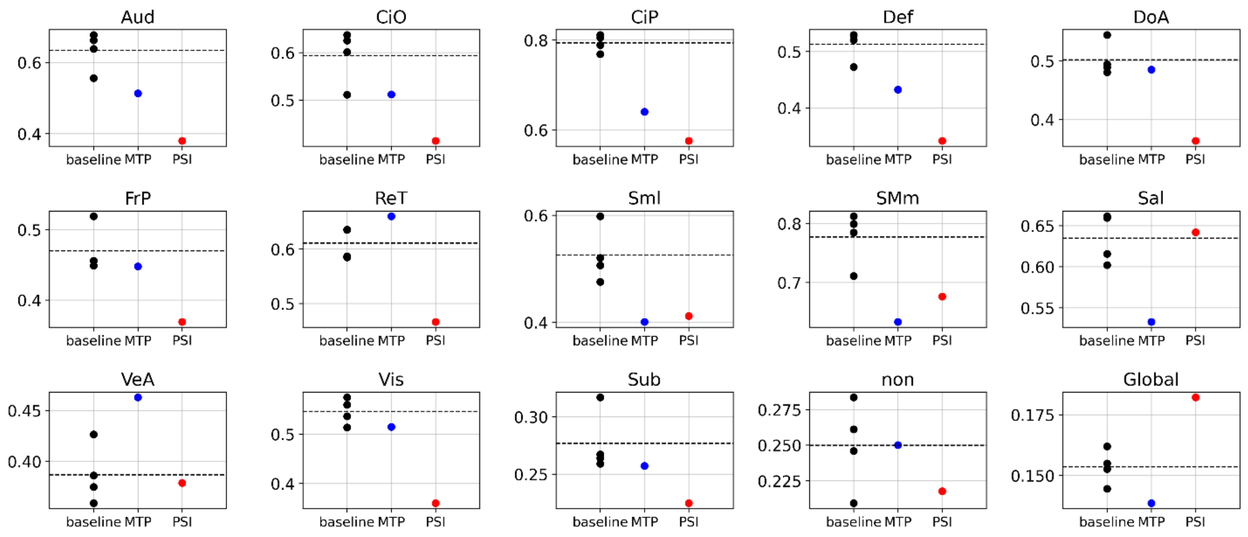

Figure S12: Sync values for all the individual functional networks for subject P4, for the different sessions: baseline (grey), methylphenidate (blue) and psilocybin (red). Abbreviations for networks labels: Auditory (Aud), Cingulo Opercular (CiO), Cingulo Parietal (CiP), Dorsal Attention (DoA), Default Mode (Def), Fronto-Parietal (FrP), Retrosplenial (ReT), Somatomotor - lateral (Sml), Somatomotor - medial (SMm), Salience (Sal), Ventral Attention (VeA), Visual (Vis), No assignment (non), Global (whole brain).

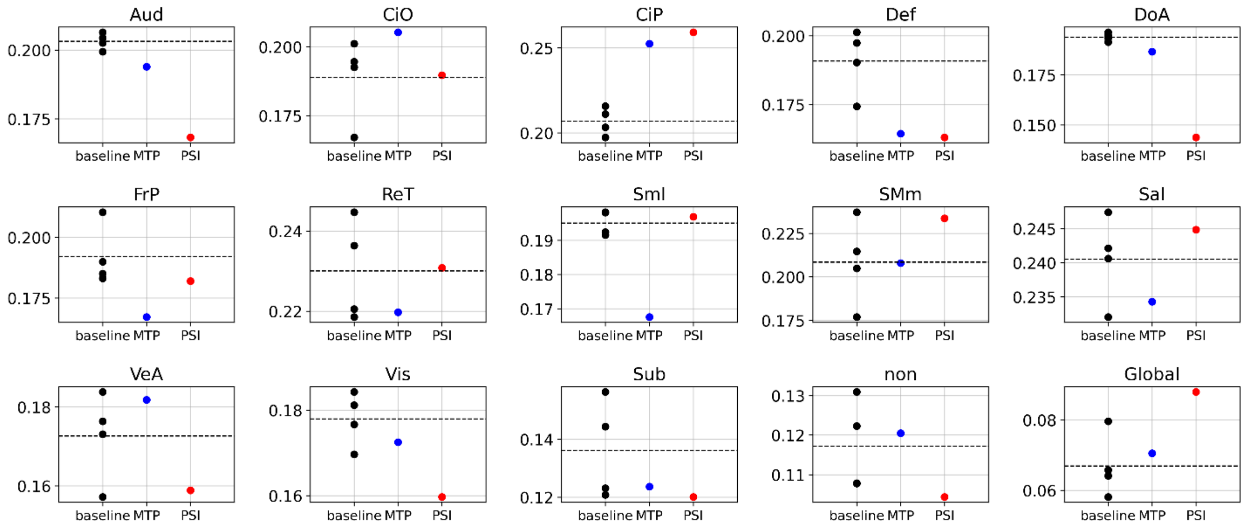

Figure S13: Same as in figure S12 but for the metastability values for subject P4

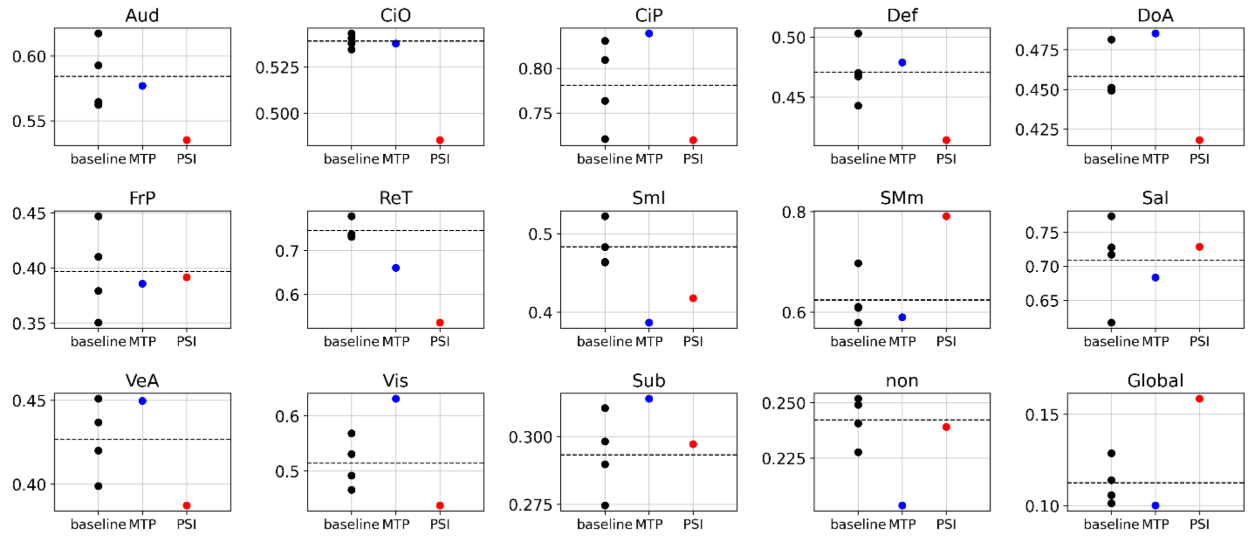

Figure S14: Sync values for all the individual functional networks for subject P6, for the different sessions: baseline (grey), methylphenidate (blue) and psilocybin (red). Abbreviations for networks labels: Auditory (Aud), Cingulo Opercular (CiO), Cingulo Parietal (CiP), Dorsal Attention (DoA), Default Mode (Def), Fronto-Parietal (FrP), Retrosplenial (ReT), Somatomotor - lateral (Sml), Somatomotor - medial (SMm), Salience (Sal), Ventral Attention (VeA), Visual (Vis), No assignment (non), Global (whole brain).

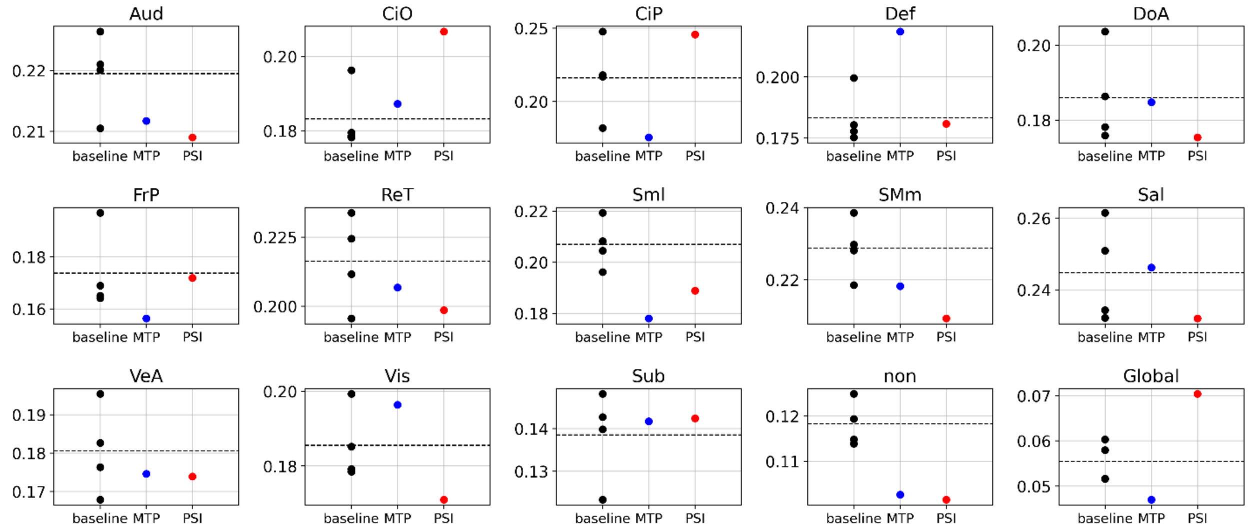

Figure S15: Same as in figure S14 but for the metastability values for subject P6.
